# Systematic review of the use of granulocyte–macrophage colony-stimulating factor in patients with advanced melanoma

**DOI:** 10.1007/s00262-016-1860-3

**Published:** 2016-07-02

**Authors:** Christoph Hoeller, Olivier Michielin, Paolo A. Ascierto, Zsolt Szabo, Christian U. Blank

**Affiliations:** 1 Department of Dermatology, Medical University Vienna, Waehringer Guertel 18–20, 1090 Vienna, Austria; 2 Department of Oncology, Lausanne University Hospital, Champ de l’Air, Rue du Bugnon 21, 1011 Lausanne, Switzerland; 3 Ludwig Centre and Swiss Institute of Bioinformatics, Génopode Building, 1015 Lausanne, Switzerland; 4 Istituto Nazionale Tumori, Fondazione ‘G. Pascale’, Via Mariano Semmola, 52, 80131 Naples, Italy; 5 Clinical Development, Amgen Europe GmbH, Dammstrasse 23, 6300 Zug, Switzerland; 6 Division of Immunology, Netherlands Cancer Institute, Plesmanlaan 121, 1066 CX Amsterdam, Netherlands

**Keywords:** GM-CSF, Granulocyte–macrophage colony-stimulating factor, Melanoma, Immunotherapy, Efficacy

## Abstract

Several immunomodulatory checkpoint inhibitors have been approved for the treatment of patients with advanced melanoma, including ipilimumab, nivolumab and pembrolizumab. Talimogene laherparepvec is the first oncolytic virus to gain regulatory approval in the USA; it is also approved in Europe. Talimogene laherparepvec expresses granulocyte–macrophage colony-stimulating factor (GM-CSF), and with other GM-CSF-expressing oncolytic viruses in development, understanding the clinical relevance of this cytokine in treating advanced melanoma is important. Results of trials of GM-CSF in melanoma have been mixed, and while GM-CSF has the potential to promote anti-tumor responses, some preclinical data suggest that GM-CSF may sometimes promote tumor growth. GM-CSF has not been approved as a melanoma treatment. We undertook a systematic literature review of studies of GM-CSF in patients with advanced melanoma (stage IIIB–IV). Of the 503 articles identified, 26 studies met the eligibility criteria. Most studies investigated the use of GM-CSF in combination with another treatment, such as peptide vaccines or chemotherapy, or as an adjuvant to surgery. Some clinical benefit was reported in patients who received GM-CSF as an adjuvant to surgery, or in combination with other treatments. In general, outcomes for patients receiving peptide vaccines were not improved with the addition of GM-CSF. GM-CSF may be a valuable therapeutic adjuvant; however, further studies are needed, particularly head-to-head comparisons, to confirm the optimal dosing regimen and clinical effectiveness in patients with advanced melanoma.

**Electronic supplementary material:**

The online version of this article (doi:10.1007/s00262-016-1860-3) contains supplementary material, which is available to authorized users.

## Introduction

Malignant melanoma accounts for approximately 1 % of cancer deaths worldwide, equating to over 55,000 deaths from this tumor type in 2012 [1]. Guidelines for the treatment of cutaneous melanoma recommend surgical resection for localized disease; however, for metastatic disease, systemic therapy is often required [2, 3]. The treatment landscape for advanced or metastatic melanoma has recently changed. Previously, patients received chemotherapy or, in some cases, IL-2 [4, 5]. In Europe, the use of IL-2 is usually recommended only in the context of a clinical trial; however, in patients with metastatic disease who do not have access to a clinical trial program or targeted therapy, IL-2 can be prescribed [3]. In contrast, in the USA, IL-2 is approved for the treatment of patients with metastatic melanoma [6]. The introduction of novel targeted therapies, such as BRAF and MEK inhibitors, and immunomodulatory checkpoint inhibitors, has improved outcomes for patients with advanced disease [7].

Several immunomodulatory agents work by overriding checkpoints in the cancer-immunity cycle, thus promoting the elimination of tumor cells by the immune system [8]. Ipilimumab, a monoclonal antibody that targets the CTLA-4 receptor, and the antibodies nivolumab and pembrolizumab, both of which target PD-1, have been approved in the USA and Europe [9–14]. In October 2015, ipilimumab was approved in the USA for adjuvant therapy in the treatment of patients with cutaneous melanoma with pathologic involvement of regional lymph nodes of more than 1 mm in diameter who have undergone complete resection, including total lymphadenectomy [9]. Ipilimumab has also been granted accelerated approval in the USA for use in combination with nivolumab for the treatment of patients with unresectable or metastatic wild-type *BRAF V600* tumors [11]. Antibodies against PD-L1 (durvalumab [15], avelumab [16] and atezolizumab [17]) are in clinical development.

More recently, oncolytic viruses expressing GM-CSF have been developed, and in 2015, talimogene laherparepvec, a modified herpes simplex virus type 1, became the first oncolytic virus to gain regulatory approval in the USA, where it is indicated for the local treatment of unresectable cutaneous, subcutaneous (s.c.) and nodal lesions in patients with melanoma recurrent after initial surgery. It has not been shown to improve overall survival (OS) or to have an effect on visceral metastases [18]. Talimogene laherparepvec is also approved in Europe for the treatment of adults with unresectable stage IIIB–IVM1a melanoma that is regionally or distantly metastatic with no bone, brain, lung or other visceral disease [19]. Talimogene laherparepvec has a proposed dual mechanism of action: The introduction of oncolytic viral particles directly into the tumor causes tumor cell lysis and local expression of the gene encoding GM-CSF induces a systemic immune response [20]. There is evidence that the virus causes regression of both the injected and uninjected lesions [21, 22]. In early preclinical studies of talimogene laherparepvec, anti-tumor responses were observed following injection of viruses with and without GM-CSF, but responses in non-injected tumors were observed only in mice that received the GM-CSF-expressing virus [22]. Other modified GM-CSF-expressing oncolytic viruses in early clinical development include JX-594 [23], CG0070 [24] and Ad5/3-D24-GMCSF [25].

In the randomized phase 3 Oncovex^GM-CSF^ Pivotal Trial in Melanoma (OPTiM), talimogene laherparepvec was compared with s.c. GM-CSF in patients with stage IIIB–IV unresected melanoma [21]. Studies of talimogene laherparepvec in combination with other agents are underway in patients with advanced melanoma, including a phase 2 study of talimogene laherparepvec in combination with ipilimumab [26] and a phase 1b/3 trial of talimogene laherparepvec in combination with pembrolizumab [27].

**Fig. 1:**
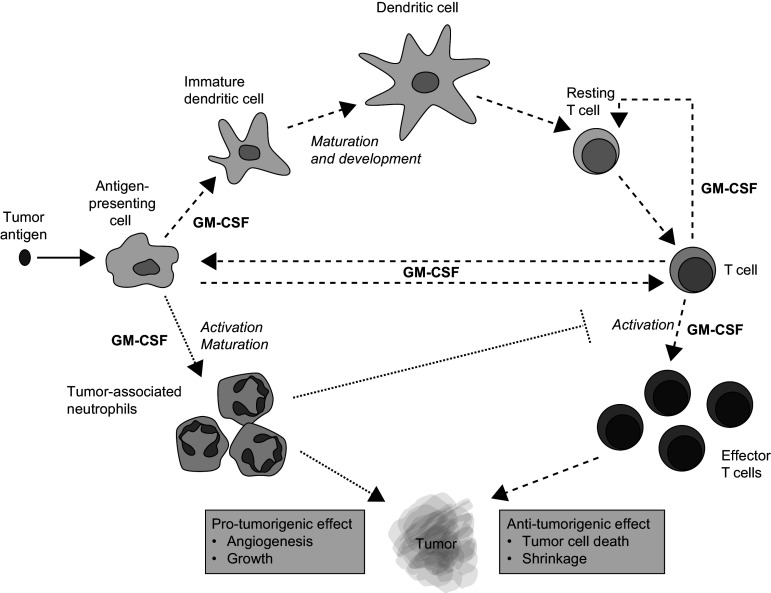
Pleiotropic role of GM-CSF in tumor immunity [28, 32, 33] GM-CSF, granulocyte–macrophage colony-stimulating factor

GM-CSF is a hematopoietic growth factor that has pleiotropic effects on the immune system (Fig. 1). It plays an important role in the development and maturation of dendritic cells (DCs) and in the activation and proliferation of T cells [28]. In response to immune stimuli, GM-CSF is produced by a variety of cell types, such as fibroblasts, epithelial cells, macrophages, T cells and tumor cells [28]. It is an important mediator of the interaction between T cells and antigen-presenting cells (APCs) and is, therefore, essential for anti-tumorigenic responses [28]. Owing to its immunobiology, GM-CSF has been investigated in clinical trials as both a monotherapy and in combination therapies. GM-CSF (sargramostim) is approved in the USA for the prevention and treatment of chemotherapy-induced neutropenia and for hematopoietic stem cell mobilization [29]. Early studies have shown that it acts as an immune adjuvant to drive humoral and cellular anti-tumor responses [28]. GM-CSF also acts as a chemoattractant for immune cells such as neutrophils [30], which can inhibit or promote tumor activity, depending on the tumor microenvironment. Tumor-associated neutrophils can induce angiogenesis [31], support tumor growth and metastases and suppress the anti-tumor immune response by decreasing the activation of CD8+ T cells [32, 33]. Therefore, in some instances, localized GM-CSF may have a detrimental effect, enabling tumor growth and progression.

One of the mechanisms of cancer immunotherapy is the protection of anti-tumor immune cells from the suppressive effects of certain cell types, such as regulatory T cells (T-regs) and myeloid-derived suppressor cells (MDSCs). MDSCs are a heterogeneous population of cells that mediate tumor-induced immune suppression. The expansion and recruitment of MDSCs is mediated by several soluble factors, including GM-CSF [34]. Therefore, there have been some concerns that therapeutic GM-CSF may have the potential to induce proliferation of these immunosuppressive cells [35]. MDSC induction has been examined in several murine tumor models. In one model, intratumoral injection of a vaccinia virus expressing GM-CSF had mixed effects on MDSCs: by itself, it increased tumor-associated, but not systemic, MDSCs; co-administration with HER2/neu, however, significantly reduced splenic and tumoral MDSCs [36]. In a model of liver metastases, expansion of MDSCs was dependent on tumor-produced GM-CSF [37]. In contrast to these findings, a different model showed that GM-CSF administered in combination with peptide vaccines caused localized accumulation of DCs and tumor-specific T cells, but no significant increase in the proportion of MDSCs [38]. In another model, tumor infiltration by MDSCs was directly correlated with splenic GM-CSF transcript levels [39]. There is somewhat limited clinical evidence for GM-CSF-mediated induction of MDSCs in patients with melanoma. In a phase 2 study of patients with stage IIIC and IV melanoma, talimogene laherparepvec-treated lesions had lower levels of MDSCs than did specimens derived from patients who had undergone surgical resection [40]. In the same study, analysis of tumor-infiltrating lymphocytes using a MART-1 ELIspot assay showed that talimogene laherparepvec induced a local antigen-specific effector T-cell response and systemic immunity against melanoma antigens. In another study, patients receiving GM-CSF in combination with peptide vaccines and TLR-9 showed that there was no significant change from baseline in the proportion of circulating immunosuppressive cells (T-regs or MDSCs) after treatment [41]. In a prospective trial, no change in MDSCs was observed in patients who received GM-CSF following surgical resection [42].

There is some evidence to suggest that the dose of GM-CSF is important in determining immune activation, but other regulatory mechanisms may underlie this process [43]. In normal conditions, GM-CSF induces milk fat globule epidermal growth factor protein 8 (MFG-E8), enabling APCs to phagocytose apoptotic cells, which in turn promotes immune tolerance and may attenuate any anti-tumor responses [44]. In some tumor microenvironments, loss of immune homeostasis and downregulation of MFG-E8 can occur. In this situation, GM-CSF no longer induces tolerance through MFG-E8 and can instead elicit an anti-tumor response [44]. Therefore, MFG-E8 may act as a regulator of GM-CSF function.

There are few studies reporting data from direct comparisons between GM-CSF and other treatments in patients with advanced melanoma. Therefore, we conducted a systematic review of the available evidence on efficacy, immunological effects and safety of GM-CSF in adult patients with stage IIIB–IV melanoma.

## Methods

### Literature search

We systematically reviewed published English language studies according to a pre-specified protocol. We searched Embase (January 1, 2000–May 1, 2015) and PubMed (January 1, 2000–May 1, 2015). Abstracts from the annual congresses of the following organizations were searched for the period January 1, 2010–April 24, 2015: American Society of Clinical Oncology, European Association of Dermato-Oncology and Society for Melanoma Research. Complete search strings are listed in electronic supplementary material.

### Inclusion/exclusion criteria

Studies were included if they enrolled adults (≥18 years old) with advanced melanoma (defined as stage IIIB–IV) who received treatment with GM-CSF as monotherapy, as part of combination therapy or in an adjuvant setting. Studies that used modified viruses, cell lines, plasmid DNA or complementary DNA (cDNA) to deliver GM-CSF were excluded. Studies of prevention or detection of melanoma, or those that included patients with non-cutaneous melanoma (e.g., ocular or mucosal melanoma), were also excluded.

### Screening and data extraction

The systematic review process described here is compliant with the 2009 preferred reporting items for systematic reviews and meta-analyses (PRISMA) guidelines [45]. The titles and abstracts of studies identified in the initial search were screened by two independent reviewers to ascertain whether they met the pre-specified inclusion criteria. Phase 1–4 studies, single-arm studies, observational studies, systematic reviews, meta-analyses and pooled analyses were included, but editorials, letters, case reports, guidelines, health technology assessment reports, economic evaluations, narrative reviews and research protocols were excluded from the final analyses.

For all studies that were deemed eligible for inclusion, and for those for which eligibility remained uncertain following the title/abstract screen, the full texts of the study manuscripts were reviewed by two independent reviewers to confirm or refute their eligibility. Data were extracted from full-text publications when available. Data from congress abstracts were included unless the same data had also been published in a peer-reviewed manuscript, in which case only the latter was included in the analysis. A protocol amendment was made at this stage and studies in which fewer than 10 patients received GM-CSF were excluded to focus the analysis on larger studies.

### Data analyses

All data were presented as reported by the authors of the publications analyzed. Mean or median values reported in the studies were used as a basis for summarizing the efficacy, immunological effects and safety of GM-CSF.

## Results

### Systematic literature search

**Fig. 2:**
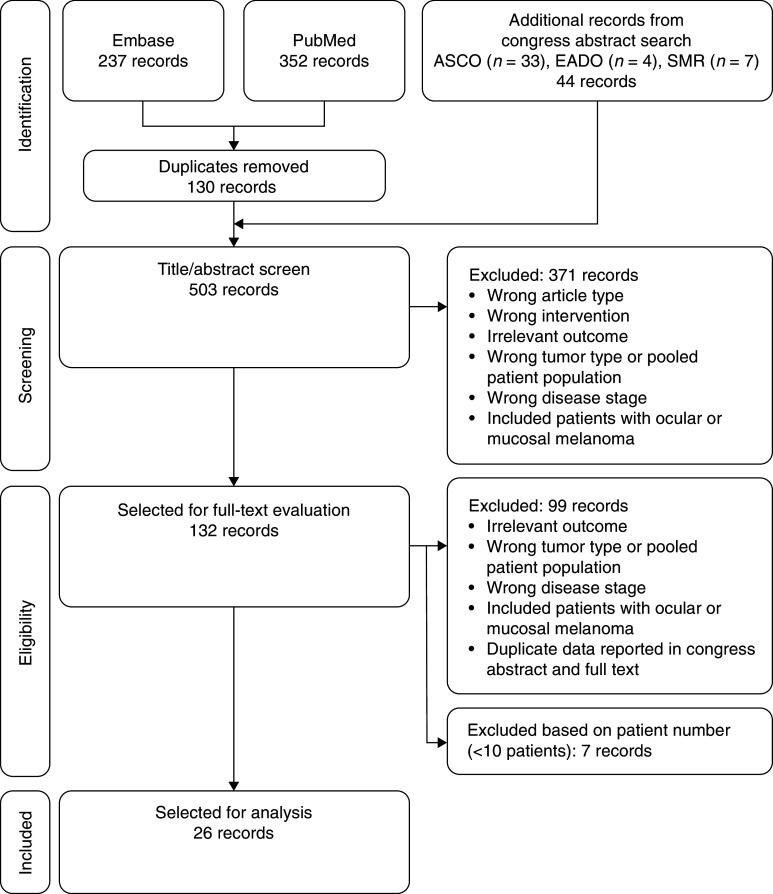
Preferred reporting items for systematic reviews and meta-analyses flow diagram ASCO, American Society of Clinical Oncology; EADO, European Association of Dermato-Oncology

We identified 589 full-text articles and 44 congress abstracts (Fig. 2). Following removal of duplicates, 503 records were analyzed based on title and abstract. At this stage, 371 studies were excluded. The full texts of the remaining articles were screened and a further 106 were excluded, leaving 26 records for analysis. Most were phase 2 studies (*n* = 16), and there were six studies that actively compared GM-CSF with other treatments and three that made comparisons with historical controls.

The study designs and dosing schedules are described in Table 1.

**Table 1:** Overview of study designs and GM-CSF dosing schedules

Author (year)	Study phase/type	Number of patients enrolled	GM-CSF dosing regimen
*Comparator studies (GM-CSF compared with non-GM-CSF treatment)*			
Schaed et al. [51]	Phase not specified; randomized	31	40 μg i.d. at a single site for 10 days in combination with peptide vaccine
Hersey et al. [48]	Phase 1/2	36	400 μg s.c.; Q2W in combination with peptide vaccine for six vaccinations
Markovic et al. [50]	Phase 2; randomized	25	10 or 50 μg s.c. in combination with peptide vaccine; Q3W for eight cycles, then every 3 months for up to 1 year
Celis et al. [46]	Phase 2; randomized	28	75 or 100 μg s.c. or no GM-CSF; in combination with peptide vaccine for up to nine vaccinations or until PD, excessive toxicity or patient refusal
Grotz et al. [47]	Retrospective cohort study	317	250 μg s.c. every day for 14 days of each 28-day cycle as an adjuvant to surgery; treatment continued for 1–3 years or until recurrence
Hodi et al. [49]	Phase 2; randomized	245	250 μg s.c. in combination with ipilimumab on days 1–14 of each 21-day cycle
*Historical comparator studies*			
Spitler et al. [54]	Phase 2	51	125 μg/m^2^ s.c. as an adjuvant to surgery for 14 consecutive days of each 28-day cycle; treatment was continued for ≥1 year or until disease recurrence or significant toxicity
O’Day et al. [53]	Phase 2	33	125 μg/m^2^ s.c. on days 1–14 (or 3–17 during pulsed cycles) for 12 cycles (28 days per cycle) following biochemotherapy
*Comparator studies (GM-CSF used in all arms)*			
Slingluff Jr et al. [69]	Phase 2	39	110 μg i.d. and s.c. in combination with peptide vaccine on days 1, 8 and 15; these injections were divided between two injection sites. On days 29, 36 and 43, one injection was given at the primary vaccination site only
Slingluff Jr et al. [70]	Phase 2; randomized	175	110 μg i.d. and s.c. in combination with peptide vaccine on days 1, 8 and 15. On days 29, 36 and 43, one injection was given at the primary vaccination site. Treatment was continued as six cycles of booster vaccinations Q3W for up to 2 years
*Single-arm studies*			
Scheibenbogen et al. [63]	Phase 2	18	75 or 150 μg i.d. and s.c. on days 1–4 and repeated in weeks 2, 4 and 6 in combination with peptide vaccines. If PD was not observed at week 10, two more vaccines were given in week 10 and week 14
Groenewegen et al. [58]	Phase 1/2	32	2.5 μg/kg s.c. on days 2–12 following chemotherapy
Weber et al. [61]	Phase 2	31	Biochemotherapy: 125 μg/m^2^ (maximum dose 250 μg) s.c. on days 6–17 of each 28-day cycle for up to eight cycles or beyond at the discretion of the treating physician
Fruehauf et al. [57]	Not specified	10	250 mg/m^2^ s.c. on days 2–12 Q2W in combination with chemotherapy
Boasberg et al. [56]	Not specified	54	Maintenance biotherapy: 125 μg/m^2^ s.c. on days 1–14 of each cycle (treatment began 4 weeks after the first day of each qualifying patient’s last cycle of concurrent biochemotherapy)
Pilla et al. [68]	Phase 2	38	75 μg s.c. on days −1, 0 and +1 for cycles 1 and 2, then administered Q2W at the same time as a peptide vaccine
Bins et al. [64]	Phase 1	11	100 μg s.c. in combination with tetanus toxoid and peptide vaccines weekly for 4 weeks
Daud et al. [42]	Phase 2	42	125 μg/m^2^ s.c. on days 1–14 of each 28-day cycle (maximum 13 cycles), as an adjuvant to surgery
Weide et al. [67]	Phase 1/2	15	150 μg s.c. 24 h after mRNA injection in weeks 0, 2, 4 and 6, then Q4W until week 34
Dillman et al. [66]	Phase 2	56	500 μg s.c. weekly for 3 weeks, then monthly for 5 months (for up to a total of 6 months or eight doses) in combination with autologous DCs
O’Day et al. [62]	Phase 2	133	Concurrent biotherapy: 500 μg i.v. on days 6–16 or until ANC ≥5000/μL in each 21-day cycle. Maintenance biotherapy: 250 μg s.c. on days 1–14 every 28 days for 12 cycles
Spitler et al. [55]	Not specified	102	125 μg/m^2^ s.c. on days 1–14 of each 28-day cycle; treatment continued for ≥3 years or until unresectable recurrence, as an adjuvant to surgery
Gunturu et al. [59]	Phase 2	20	250 μg/m^2^ s.c. daily from day 8 until ANC recovery, following chemotherapy
Locke et al. [60]	Phase 2	20	250 μg/m^2^ s.c. on days 3–12 following chemotherapy or until WBC count recovery, whichever occurred first. Treatment cycles continued Q3W until progression or toxicity
Adamina et al. [65]	Phase 1/2	16	5 μg/kg s.c. every 5 days in each 7-day cycle, alternating between a week of treatment and a week of rest over two 7-week courses, in combination with vaccinia virus
Eroglu et al. [52]	Phase 2	52	250 mg/m^2^ s.c. on days 2–12 Q2W in combination with chemotherapy

*ANC* absolute neutrophil count, *PD* progressive disease, *Q2W/Q3W/Q4W* every 2/3/4 weeks

### Comparative studies

Overall, six of the included studies made direct comparisons between GM-CSF treatment and non-GM-CSF treatments [46–51], and three studies compared patients treated with GM-CSF with historical controls who did not receive GM-CSF [52–54].

#### GM-CSF in combination with systemic therapy

Significant improvements in OS in patients who received GM-CSF treatment as an adjuvant to systemic therapy were reported in two studies. A phase 2 randomized study reported significantly prolonged median OS when GM-CSF was added to ipilimumab compared with ipilimumab alone (17.5 vs. 12.7 months; *P* = 0.01) [49]. The 1-year OS was also significantly improved in the GM-CSF group compared with the group receiving ipilimumab alone (1-year OS: 68.9 vs. 52.9 %; *P* = 0.01), but median progression-free survival (PFS) was not significantly different between treatment arms (3.1 months each for the GM-CSF group and the ipilimumab group; *P* = 0.37) [49]. The response rates were similar between groups: 15.5 and 14.8 % in the GM-CSF and ipilimumab-alone groups, respectively [49]. In this trial, significantly fewer grade 3–5 adverse events were reported in patients receiving GM-CSF in combination with ipilimumab than in those receiving ipilimumab alone (44.9 vs. 58.3 %; *P* = 0.04); however, there were high numbers of deaths in both treatment groups. The improvements in OS observed in the GM-CSF group may not necessarily be due to increased efficacy when GM-CSF is added to ipilimumab. Reduced toxicity and the resulting lower treatment dropout rate in the GM-CSF arm may have contributed to the observed OS improvement, but this remains to be determined. Furthermore, it is unclear whether treatment discontinuation in the ipilimumab group was due to adverse events or other reasons, such as disease progression [49].

In another phase 2 study, patients who received a novel GM-CSF-containing maintenance biochemotherapy regimen after induction biochemotherapy had significantly longer median OS than historical controls who had received an identical biochemotherapy regimen without GM-CSF (18.5 vs. 9.3 months; *P* = 0.0004) [53]. The median PFS was also significantly prolonged (GM-CSF, 8.1 months; historical controls, 5.9 months; *P* = 0.0015), and multivariate analyses showed that maintenance biotherapy was predictive of PFS and OS [53].

Eroglu et al. [52] investigated the use of GM-CSF in combination with docetaxel and vinorelbine. The 1-year OS was 48.1 %, which was significantly higher than the predicted survival according to the Korn analysis for this patient group (24.3 %; *P* = 0.012) [52].

#### GM-CSF in combination with peptide vaccines

GM-CSF used in combination with peptide vaccines had no significant effect on survival in two randomized phase 2 studies. In one study, median OS values for patients receiving no GM-CSF, GM-CSF 75 μg or GM-CSF 100 μg were 10, 6 and 9 months, respectively. This peptide vaccine was deemed clinically ineffective [46]. The other study reported that the addition of low-dose GM-CSF to melanoma peptide (MP) vaccines emulsified in Montanide ISA-51 did not improve median OS or median PFS [50]. In a phase 1/2 study, no clinical responses were reported in patients receiving T-cell peptide epitopes with or without GM-CSF [48].

#### GM-CSF in the adjuvant setting

In studies that included patients who received adjuvant GM-CSF following complete surgical resection, most of the participants had stage IIIB or higher disease [47, 54, 55]. In a retrospective cohort study by Grotz et al. [47], adjuvant GM-CSF use was not associated with a significant difference in disease-free or melanoma-specific survival (MSS); however, within a subgroup of patients with stage IIIC disease, a 52 % lower melanoma-specific mortality was reported in individuals receiving GM-CSF than in those who were under observation only (hazard ratio 0.48; *P* = 0.02). The low patient numbers and lack of important information on patient characteristics, such as mutation status or subsequent therapy, make the interpretation of this data set difficult.

Spitler et al. [54] evaluated GM-CSF as an adjuvant therapy in patients who were at high risk of recurrence. Individuals had to have stage III disease with more than four positive nodes and a tumor larger than 3 cm in diameter. Median OS was significantly prolonged in those who received GM-CSF compared with matched historical controls (37.5 vs. 12.2 months; *P* = 0.001). The 1-year OS and 2-year OS were also significantly higher in the GM-CSF group than in the control group (89 vs. 45 % [*P* = 0.001] and 64 vs. 15 % [*P* = 0.001], respectively) [54].

In another study, prolonged GM-CSF treatment was given to patients for at least 3 years following surgical resection. The 5-year MSS rates were 67 and 40 % for patients with stage III and IV disease, respectively [55]. The majority (62/98) of patients had one or more disease recurrences, of which over half were successfully excised [55].

### Single-arm studies

Overall, 16 studies in which GM-CSF was not compared with another treatment arm were included. These are summarized in Table 2.

**Table 2:** Overview of single-arm studies

Author (year)	Study phase	Number of patients enrolled	Treatment and GM-CSF dosing regimen	Clinical outcomes
Groenewegen et al. [58]	Phase 1/2	32	Sequential treatment: DTIC 800 mg/m^2^ i.v. on day 1, followed by GM-CSF 2.5 µg/kg s.c. on days 2–12, IL-2 1.8 × 10^6^ units on days 8–18 and IFN-α 6 × 10^6^ units s.c. on days 15–20	Median OS: 244 days 1-year OS: 22 % 2-year OS: 12 % ORR (95 % CI): 32 % (16–49 %) CR: 13 % PR: 19 %
Adamina et al. [65]	Phase 1/2	16	In combination with recombinant vaccinia virus and soluble peptide: intranodal vaccinia virus given on days 3 and 59; peptides given on days 17, 31, 45, 73, 87 and 101. GM-CSF 5 µg/kg s.c. given for 5 days, starting on the day of the intranodal injections	Median OS: 488.5 days Mean OS (responsive patients): 1106 days Mean OS (non-responsive patients): 696 days Median PFS: 310 days Mean PFS (responsive patients): 547 days Mean PFS (non-responsive patients): 338 days
Scheibenbogen et al. [63]	Phase 2	18	In combination with peptide vaccine: GM-CSF 75 or 150 µg i.d. and s.c. at the same site on days 1 and 4. Identical vaccinations were repeated at weeks 2, 4 and 6. If PD not observed, additional vaccinations were administered in weeks 10 and 14	Stable disease: 2 Mixed response: 1 NED: 2^a^ PD: 13
Pilla et al. [68]	Phase 2	38	In combination with peptide vaccine: weekly vaccine for 4 weeks, starting 5–8 weeks after surgery. If no PD after 4 weeks, patients received four injections Q2W. GM-CSF 75 µg s.c. was given on days −1, 0 and 1 and then Q2W at the same time as the vaccine. IFN-α 3 × 10^6^ units s.c. was given on days 1 and 3 after the last administration of GM-CSF during the first cycle. During the second cycle, IFN-α was given three times per week during the weeks in between vaccinations	Median OS (95 % CI): 583 days (291 days–NR) Time to progression (95 % CI): 145 days (97–188 days) CR: 5 % Stable disease: 55 % PD: 40 %
Bins et al. [64]	Phase 1	11	In combination with peptide vaccine and gp100: GM-CSF 100 µg s.c. weekly for 4 weeks	Median OS: 4 months ORR: 0 %
Boasberg et al. [56]	Phase not specified	54	Biochemotherapy: DTIC, cis-platinum and vinblastine i.v., IFN-α s.c. and IL-2 i.v. with decrescendo dosing. Maintenance biotherapy: low-dose IL-2 (1 MIU/m^2^) + GM-CSF 125 µg/m^2^ s.c. on days 1–14 of each cycle (treatment began 4 weeks after the first day of the qualifying patient’s last cycle of biochemotherapy)	Median OS (95 % CI) (vitiligo): 18.2 months (12.3 months–NR) Median OS (95 % CI) (no vitiligo): 8.5 months (6.7–12.7 months) *P* = 0.027
Daud et al. [42]	Phase 2	42	Adjuvant to surgery: GM-CSF 125 µg/m^2^ s.c. on days 1–14 of each 28-day cycle (maximum 13 cycles)	Median OS (95 % CI): 65.3 months (47.4–67.0 months) Median recurrence-free survival (95 % CI): 5.6 months (3.5–11.7 months)
Spitler et al. [55]	Phase not specified	102	Adjuvant to surgery: GM-CSF 125 µg/m^2^ s.c. on days 1–14 of each 28-day cycle for at least 3 years or until unresectable recurrence	5-year MSS (95 % CI) (stage III): 67 % (56–79 %) 5-year MSS (95 % CI) (stage IV): 40 % (19–60 %)
Weide et al. [67]	Phase 1/2	15	In combination with autologous mRNA: GM-CSF 150 µg s.c. 24 h after mRNA injection in weeks 0, 2, 4 and 6, then Q4W until week 34	Stable disease: 1 PD: 9 NED: 3 Mixed response: 2
Weber et al. [61]	Phase 2	31	In combination with chemotherapy: cycle 1 daily oral temozolomide 150–200 mg/m^2^ for 5 days followed by biotherapy (GM-CSF 125 µg/m^2^ up to a maximum dose of 250 µg/m^2^ s.c. + IFN 5 × 10^6^ units + IL-2 4 × 10^6^ units/m^2^) daily for 12 days. This 28-day cycle was repeated as clinically indicated	Median OS (95 % CI): 13.1 months (7.8–18.3 months) 1-year OS: 52 % 2-year OS: 25 % Median PFS (95 % CI): 4.9 months (2.8–6.9 months) 1-year PFS: 29 % 2-year PFS: 10 % ORR (95 % CI): 26 % (12–45 %) CR: 4 (2 NED) PR: 4 Stable disease: 7 Overall benefit rate (95 % CI): 48 % (30–67 %)
Eroglu et al. [52]	Phase 2	52	In combination with chemotherapy: docetaxel and vinorelbine every 14 days, followed by GM-CSF 250 mg/m^2^ s.c. on days 2–12 of each cycle	Median OS (95 % CI): 320 days (190–390 days) 1-year OS: 48.1 % (historical control OS 24.3 %; *P* = 0.012) Median PFS (95 % CI): 134 days (91–214 days) ORR: 15.4 % CR: 0 % PR: 15.4 % Stable disease: 36.5 % PD: 30.7 % Clinical benefit rate: 52 %
Gunturu et al. [59]	Phase 2	20	In combination with chemotherapy: cyclophosphamide and fludarabine followed by two 5-day courses of high-dose IL-2 6 × 10^6^ units/kg i.v. on days 8–12 and 21–25. GM-CSF 250 µg/m^2^ s.c. per day from day 8 until granulocyte recovery	Median OS: 1.1 years^b^ Median PFS: 0.25 years^b^ ORR: 22.2 % CR: 5.6 % PR: 16.7 %
Locke et al. [60]	Phase 2	20	In combination with chemotherapy: docetaxel, oxaliplatin, dexamethasone and ondansetron. GM-CSF 250 µg/m^2^ s.c. on days 3–12 or until WBC count recovery, whichever comes first; treatment continued Q3W until progression or toxicity	Median OS: 5.4 months (range, <1–17 months^c^) Median PFS: 1.4 months (range, <1–8 months^c^) ORR: 0 % Stable disease: 26.3 % PR: 64.3 %
Fruehauf et al. [57]	Phase not specified	10	In combination with chemotherapy: docetaxel and vinorelbine. GM-CSF 250 mg/m^2^ s.c. on days 2–12 of each cycle	ORR: 50 % PR: 50 % Median time to progression: 8 months
O’Day et al. [62]	Phase 2	133	Biochemotherapy: DTIC, cis-platinum and vinblastine, IFN-α s.c. and IL-2 i.v. with decrescendo dosing plus GM-CSF 500 µg s.c. for 10 days. Maintenance biotherapy: low-dose IL-2 (1 MIU/m^2^) + GM-CSF 125 µg/m^2^ s.c. on days 1–14 of each cycle	Median OS (95 % CI): 13.5 months (11.5–15.4 months) 1-year OS (±SE): 56.5 % ± 4.0 2-year OS (±SE): 23 % ± 3.7 Median PFS (95 % CI): 9 months (7.8–12.0 months) Estimated 1-year PFS (±SE): 40.9 % ± 4.3 Estimated 2-year PFS (±SE): 15.9 % ± 3.2
Dillman et al. [66]	Phase 2	56	In combination with autologous DC vaccine: vaccine suspended in GM-CSF 500 µg before s.c. administration weekly for 3 weeks, then monthly for 5 months for up to 6 months or a maximum of eight doses	Median OS: NR 1-year OS: 85 % 2-year OS: 72 % Predicted 5-year OS: 54 % Median PFS: 4.2 months 5-year PFS: 23 % ORR: 0 % Stable disease: 40 % PD: 60 %

*CI* confidence interval, *CR* complete response, *DTIC* dacarbazine; *gp100* glycoprotein-100, *MIU* million international unit, *MSS* melanoma-specific survival, *NED* no evidence of disease, *NR* not reached, *ORR* overall response rate, *PD* progressive disease, *PFS* progression-free survival, *PR* partial response, *Q2W/Q3W/Q4W* every 2/3/4 weeks, *SE* standard error

^a^Two patients had complete surgical resection before study entry and therefore remained with NED throughout

^b^Medians estimated from Kaplan–Meier curves

^c^Range for deceased patients

#### GM-CSF in combination with chemotherapy

Several single-arm studies evaluated the use of GM-CSF in combination with chemotherapy [52, 56–62]. When docetaxel, vinorelbine and GM-CSF were administered in a study conducted by Fruehauf et al., the overall response rate (ORR) was 50 % and the median time to progression was 8 months [57]. In a similarly designed study, Eroglu et al. [52] reported an ORR of 15.4 %. Median OS and PFS were 320 and 134 days, respectively, and the clinical benefit rate was 52 % [52]. In another study, cyclophosphamide and fludarabine were followed by high-dose IL-2 and GM-CSF to facilitate granulocyte recovery [59]. The ORR was 22.2 % and estimated median OS and PFS were 1.1 and 0.25 years, respectively [59]. Weber et al. [61] administered daily oral temozolomide followed by biotherapy (GM-CSF in combination with IFN and IL-2) and reported an ORR of 26 %. The median PFS was 4.9 months and the overall benefit rate was 48 % [61]. In a phase 1/2 study, dacarbazine (DTIC) followed by GM-CSF, IL-2 and IFN-α resulted in an ORR of 32 % [58].

Two studies assessed biochemotherapy followed by maintenance biochemotherapy [56, 62]. Boasberg et al. stratified survival according to whether or not patients developed vitiligo: Median OS was 18.2 months in patients who developed vitiligo during treatment compared with 8.5 months in those who did not (*P* = 0.027) [56]. O’Day et al. [62] used a similar treatment regimen, but with a higher dose of GM-CSF, and reported a median OS of 13.5 months.

#### GM-CSF in combination with peptide vaccines

Scheibenbogen et al. [63] administered GM-CSF at the same site as a peptide vaccine and reported limited clinical efficacy. In another study, no objective responses were reported when GM-CSF was given in combination with a peptide vaccine [64]. More promising results were obtained in a phase 1/2 trial of an intranodal injection of recombinant vaccinia virus followed by soluble peptides and GM-CSF [65]. Mean OS was 1106 days in responsive patients and 696 days in non-responsive patients [65].

#### GM-CSF in combination with other treatments

In a phase 2 trial of GM-CSF in combination with an autologous DC vaccine, 1-year OS and 5-year predicted OS were 85 and 54 %, respectively [66]. Despite this, no objective responses were recorded [66]. Weide et al. [67] reported no objective responses when autologous mRNA derived from individual metastasizing tumors was administered to patients.

#### GM-CSF in the adjuvant setting

In a phase 2 trial, patients with stage IV disease who had undergone surgical resection were given a peptide vaccine and adjuvant GM-CSF followed by IFN-α. After one treatment cycle, 55 % of patients had stable disease and 5 % were disease-free. The median OS was 583 days [68]. Two studies with similar designs also used GM-CSF as an adjuvant to surgery. In one study, median OS was 65.3 months and median recurrence-free survival was 5.6 months [42]. In the other study, the 5-year MSS was 67 % for patients with stage III disease and 40 % for those with stage IV disease [55].

### Evidence of immune activation by GM-CSF

Most studies reported evidence of immune activation, and these data are summarized in Table 3. When GM-CSF was used in combination with a peptide vaccine, immunization against peptide-specific antigens was observed. In a single-arm study, proliferative immune responses to six melanoma helper peptides (MHPs) were observed in 81 % of patients [69]. In patients with stage IV disease, peptide vaccines with adjuvant GM-CSF generated CD8+ and CD4+ responses ranging from 0 to 41 % and from 5 to 47 %, respectively, across treatment groups, and there was a significant association between CD4+ response and survival (*P* = 0.0045) [70]. When immune responses were analyzed by treatment group, CD8+ and CD4+ responses were observed in 29.3 % and 16.4–21.1 % of patients, respectively, in those receiving peptide vaccines with GM-CSF and Montanide ISA-51 adjuvants [70]. Markovic et al. [50] reported twofold or greater increases from baseline in the number of tetramer-positive cytotoxic T cells for at least one vaccine-specific peptide in 37.5 % (3/8) of patients who did not receive GM-CSF and in 22.2 % (2/9) and 57.1 % (4/7) of those who received GM-CSF 10 and 50 μg, respectively. When GM-CSF was given in combination with a peptide vaccine in another phase 2 study, there were no differences in immunization efficacy across treatment arms. Therefore, addition of GM-CSF was ineffective at enhancing immunogenicity in this study [46].

**Table 3:** Evidence of immune activation by GM-CSF

Author (year)	Study phase	Number of patients enrolled	GM-CSF dosing schedule	Immunological responses
Celis et al. [46]	Phase 2	28	75 or 150 μg s.c. in combination with peptide vaccine (MPS160) for up to nine vaccinations	Immunization against MPS160: 57 % MPS160 + Montanide ISA-51: 66.7 % (6/9 patients) MPS160 + Montanide ISA-51 + GM-CSF 75 μg: 60 % (3/5 patients) MPS160 + Montanide ISA-51 + GM-CSF 100 μg: 42.9 % (3/7 patients)
Hersey et al. [48]	Phase 1/2	36	400 μg s.c. in combination with peptide vaccine Q2W for six vaccinations	DTH GM-CSF: 52.9 % (9/17 patients) No GM-CSF: 17.6 % (3/17 patients) Injection site reactions: 64.7 % (11/17 patients)
Markovic et al. [50]	Phase 2	25	10 or 50 μg s.c. Q3W for eight cycles then every 3 months for up to 1 year following surgical resection	Proportion of patients with tetramer-positive cytotoxic T cells for at least one vaccine-specific peptide Peptide + Montanide ISA-51: 37.5 % (3/8 patients) Peptide + Montanide ISA-51 + GM-CSF 10 μg: 22.2 % (2/9 patients) Peptide + Montanide ISA-51 + GM-CSF 50 μg: 57.1 % (4/7 patients) DTH: 1 patient who received peptide + Montanide ISA-51
Hodi et al. [49]	Phase 2	245	250 μg s.c. in combination with ipilimumab on days 1–14 of each 21-day cycle	Median change in CD8 + ICOS T cells (*P* = 0.01) Ipilimumab + GM-CSF: 0.5 Ipilimumab: 0.4 Median change in CD4 + ICOS T cells (*P* = 0.11) Ipilimumab + GM-CSF: 2.55 GM-CSF: 1.85
Schaed et al. [51]	Phase not specified	31	40 μg i.d. for 10 days in combination with peptide vaccine	T-cell responses to peptide vaccine: 30.8 % (8/26 patients) Peptide + Montanide ISA-51: 0 % (0/9 patients) Peptide + QS-21: 44.4 % (4/9 patients) Peptide + GM-CSF: 50.0 % (4/8 patients) Median number of peptide vaccine-reactive T cells 8 weeks post-vaccination Peptide + Montanide ISA-51: 1/14 000 cells Peptide + QS-21: 1/3125 cells Peptide + GM-CSF: 1/4545 cells
Slingluff CL Jr et al. [70]	Phase 2	175	110 μg i.d. and s.c. in combination with peptide vaccine on days 1, 8 and 15, and 110 μg s.c. on days 29, 36 and 43	CD4 + response to 12MP 12MP group: 43 % 12MP + tetanus group: 47 % 12MP + 6MP group: 28 % 6MHP group: 5 % CD8 + response to 6MHP 12MP group: 3 % 12MP + tetanus group: 0 % 12MP + 6MP group: 40 % 6MHP group: 41 % Overall CD8 + response: 29.3 % Overall CD4 + responses: 16.4 % (tetanus peptide) and 21.1 % (6MHP)
Slingluff Jr et al. [69]	Phase 2	39	110 μg i.d. and s.c. in combination with peptide vaccine on days 1, 8 and 15, and 110 μg s.c. on days 29, 36 and 43	DTH response: 29.2 % (7/24) Proliferative immune response to 6MHP: 81 %
Bins et al. [64]	Phase 1	11	100 μg s.c. weekly for 4 weeks in combination with peptide vaccine and tetanus toxoid	CD8+ peptide-specific responses: 27.3 % (3/11 patients) DTH: 18.2 % (2/11 patients) Tetanus toxoid immunoglobulin titer increases: 90 % (9/10 patients)
Boasberg et al. [56]	Phase not specified	54	125 μg/m^2^ s.c. on days 1–14 of each cycle as part of a maintenance biochemotherapy regimen	TRP-2 antibody induction Vitiligo: 29 % No vitiligo: 14 %
Daud et al. [42]	Phase 2	42	125 μg/m^2^ s.c. on days 1–14 of each 28-day cycle (maximum 13 cycles), as an adjuvant to surgery	GM-CSF caused a transient increase in mature DCs but not myeloid-derived suppressor cells
Dillman et al. [66]	Phase 2	56	500 μg s.c. weekly for 3 weeks then monthly for 5 months for up to 6 months or eight doses in combination with autologous DCs	Positive DTH test: 22.2 % (12/54) at week 4 and/or week 24 (*P* = 0.003)
Groenewegen et al. [58]	Phase 1/2	32	2.5 μg/kg s.c. on days 2–12 following chemotherapy	Significant increases in the number of CD4+ (*P* < 0.001) and CD8+ (*P* = 0.007) cells, together with CD4+/CD28+ and of CD8+/CD28+ double-positive cells (*P* values not shown) increased significantly during treatment with GM-CSF
Gunturu et al. [59]	Phase 2	20	250 μg/m^2^ s.c. daily following chemotherapy (cyclophosphamide 60 mg/kg i.v. days 1–2; fludarabine 25 mg/kg days 3–7) from day 8 until ANC recovery. IL-2 600,000 U/kg i.v. was given in 5-day courses over days 8–12 and days 21–25	Induction of melanoma-specific T cells: 25 % (1/4 evaluable patients)
Pilla et al. [68]	Phase 2	38	75 μg s.c. on days −1, 0 and 1 for cycles 1 and 2, then Q2W in combination with tumor-derived heat-shock protein	Tumor infiltration: 42.9 % (3/7 patients) Increased T-cell reactions against autologous melanoma cells: 53.8 % (7/13 patients) Increased reactions against allogeneic melanoma cell lines: 26.7 % (4/15 patients) Increase in number of activated NK cells: 61.1 % (11/18 patients)
Scheibenbogen et al. [63]	Phase 2	18	75 μg or 150 μg i.d. and s.c. on days 1–4 in combination with peptide vaccine and repeated in weeks 2, 4 and 6. If PD not observed, additional vaccines given in weeks 10 and 14	T-cell responses: 26.7 % (4/15 patients)
Weide et al. [67]	Phase 1/2	15	150 μg s.c. in weeks 0, 2, 4 and 6, then Q4W until week 34, in combination with mRNA vaccine	Unconfirmed T-cell responses: 33.3 % (5/15 patients) Humoral immunity: 26.7 % (4/15 patients)
Adamina et al. [65]	Phase 1/2	16	5 μg/kg s.c. every 5 days in each 7-day cycle, alternating between a week of treatment and a week of rest over two 7-week courses, in combination with vaccinia virus	MART-1 and gp100 staining in in vitro stimulated CD8 + T cells: 100 % Response to TAA encoded by the recombinant vaccinia virus: 100 %

*ANC* absolute neutrophil count, *DTH* delayed-type hypersensitivity, *gp100* glycoprotein 100, *MART-1* melanoma antigen recognized by T cells 1, *MHP* melanoma helper peptide, *MP* melanoma peptide, *PD* progressive disease; *Q2W/Q3W/Q4W* every 2/3/4 weeks, *TRP-2* tyrosine-related protein 2

Adamina et al. [65] used GM-CSF as an adjuvant following intranodal injection of recombinant vaccinia virus and MPs. All patients had an immune response to the tumor-associated antigen epitope encoded by the vaccinia virus. Furthermore, all T cells were responsive to in vitro stimulation with the epitopes melanoma antigen recognized by T cells 1 (MART-1) and glycoprotein 100 (gp100) [65]. In another study, positive delayed-type hypersensitivity (DTH) reactions were observed in nine of 17 patients receiving GM-CSF with or without Montanide ISA-720 and in three of 17 patients who did not receive GM-CSF [48]. More severe hypersensitivity reactions were observed in patients receiving GM-CSF and peptides alone than in those receiving GM-CSF and peptides together with Montanide ISA-720. This suggests that GM-CSF may be beneficial only when peptides are not administered together with strong adjuvants [48].

Although signs of peptide-specific immune activation were present in most studies, it appears likely to be non-functional due to the lack of correlation with anti-tumor responses [46]. Celis [46] suggest that the phenotypic evidence of immunization in the absence of clinical activity observed in their study was a result of immune dysfunction illustrated by the abnormal cytokine profiles detected in peripheral blood. In another study, Schaed et al. [51] reported no T-cell responses in patients who received the peptide vaccine emulsified in Montanide ISA-51. In those who received the vaccine with the adjuvants QS-21 or GM-CSF, the T-cell response rates were 44.4 % (4/9) and 50.0 % (4/8) of patients, respectively. Eight weeks after immunization, the median number of T cells reactive to the peptide vaccine was highest in the QS-21 adjuvant group (1/3125 cells) followed by the GM-CSF group (1/4545 cells) and the Montanide ISA-51 group (1/14 000 cells). However, this increase was transient, and 2 weeks after completion of treatment, the median number of reactive T cells was not significantly increased in any of the treatment groups. In this study, clinical benefit was not analyzed, and the magnitude of T-cell response necessary for therapeutic effects has yet to be determined [51]. In another study in which GM-CSF was used as an adjuvant to a peptide vaccine, a cytotoxic T-cell response was observed in only 26.7 % (4/15) of patients receiving GM-CSF; these individuals did not have progressive disease (PD) at the time of analysis. Of those who did have PD, no immune reactivity against the peptide vaccine was observed. The majority of patients (61 %) discontinued the study early owing to disease progression [63].

GM-CSF enhanced the immune response to ipilimumab, as measured by activation of inducible costimulator (ICOS) T cells. In a phase 2 study evaluating ipilimumab with or without GM-CSF, the median change in CD8+ ICOS T cells was significantly greater in patients who received ipilimumab and GM-CSF than in those who received ipilimumab alone (0.5 vs. 0.4 %; *P* = 0.01) [49]. Groenewegen et al. [58] reported significant increases from baseline in the number of CD4+ (*P* < 0.001) and CD8+ (*P* = 0.007) cells and a minor increase in the number of natural killer (NK) cells when GM-CSF was used as an adjuvant to chemotherapy. In another study, in which GM-CSF was given as an adjuvant to chemotherapy, induction of melanoma-specific T cells was observed in one of four evaluable patients. GM-CSF was used to support granulocyte recovery in this study, and IL-2 was also given to patients at the same time [59].

When GM-CSF was combined with tumor-derived autologous heat-shock protein gp96, increased T-cell reactions against autologous and allogeneic melanoma cells were observed relative to baseline in 53.8 % (7/13) and 26.7 % (4/15) of patients, respectively. Increases in the number of NK cells were also observed in 61.1 % (11/18) of patients [68]. In another study of adjuvant GM-CSF following surgery, a transient increase in the levels of mature DCs in peripheral blood was observed following GM-CSF treatment, but levels normalized by 4 weeks after the start of treatment [42]. Furthermore, subsequent cycles of GM-CSF treatment did not change the levels of mature DCs in peripheral blood [42].

## Discussion

This is the first systematic review of the efficacy of GM-CSF in patients with advanced melanoma. Most studies were phase 1 or 2, but study phase was not reported in some studies; no phase 3 data were available. The study groups varied in size, but most enrolled fewer than 50 patients, and those that had multiple comparator arms often had fewer than 10 patients per treatment group. Furthermore, several studies used historical controls or calculated survival rates as comparators, which makes conclusions from these studies problematic. A range of dosing schedules and routes of administration was used for GM-CSF, but GM-CSF was most often delivered by s.c. injection at a dose of 125–250 mg/m^2^.

There was some evidence of clinical benefit in patients who received GM-CSF in combination with ipilimumab [49] or as part of a chemotherapy-containing regimen [52, 53, 56, 57, 59, 62]. Ipilimumab is an immunomodulatory checkpoint inhibitor and has an activating effect on the immune system [71]. Therefore, there may be an additive effect when GM-CSF is used with ipilimumab. Stimulation of DCs by GM-CSF upregulates costimulatory molecules that are expressed by DCs, which bind to and activate T cells; however, the presence of CTLA-4 on T cells inhibits this interaction. Simultaneous inhibition of CTLA-4 and stimulation of DCs would, therefore, be expected to enhance T-cell responses [71]. The clinical benefit observed with the addition of GM-CSF may also be attributed to a reduction in the incidence of adverse events; thus, patients are able to receive more cycles of treatment than those who do not receive it. The potential synergy between immune checkpoint inhibitors and GM-CSF is interesting and may warrant further investigation.

The role of chemotherapy in immune activation is less clear, although there is accumulating evidence to suggest that off-target effects of chemotherapy may modulate the innate and adaptive arms of the immune system [72]. Chemotherapy can deplete immune cells because it is indiscriminate in killing rapidly dividing cells, and can also modulate the immune system through several mechanisms. First, it can reduce the number of tumor-induced suppressor cells. Secondly, chemotherapy kills tumor cells, thereby increasing antigenicity and, thirdly, it may directly induce an effector response by activating T cells [72]. DTIC and vinblastine have been shown to induce DC maturation, thereby stimulating immunogenic tumor cell death [73, 74]. In one phase 2 study, the use of GM-CSF as an adjuvant to chemotherapy was shown to modulate the immune system by inducing CD4+ regulatory cells, CD8+ suppressor T cells and memory T cells following lymphodepletion [59]. Furthermore, in 25 % of evaluable patients, the circulating melanoma-specific CD8+ cell population was expanded [59]. Hence, a synergistic enhancement of the immune response may occur following administration of GM-CSF to patients receiving chemotherapy; however, many studies of chemotherapy in combination with GM-CSF did not report immunological endpoints. Therefore, it is difficult to draw conclusions on the efficiency of immune activation when these two therapies are used in combination.

Activation of the immune response may correlate with anti-tumor activity. Boasberg et al. assessed a novel biochemotherapy regimen containing GM-CSF and IL-2 and reported that median OS was significantly improved in patients exhibiting immune activation, as indicated by the development of vitiligo [75], compared with those who did not develop the condition [56]. Antibodies against tyrosinase-related protein-2, a protein highly expressed in cutaneous melanoma [76], were detected in a larger proportion of patients with vitiligo than in those without, suggesting increased autoimmunity against melanoma cells in those who developed vitiligo [56]. In another study, patients with immune activation following intranodal injection of a recombinant vaccinia virus were more likely to have a clinical response than those who did not show signs of immune activation [65].

GM-CSF in combination with peptide vaccines showed limited activity. Although T-cell activation was observed in several studies, this was not accompanied by a clinical anti-tumor response [46, 50, 63]. Furthermore, the immune induction observed may be transient, as observed in one study [51]. Although not eligible for inclusion in this systematic review, because it did not report data for our target population separately, a phase 2 randomized study of patients with stage IIB–IV resected melanoma who received a vaccine comprising 12 MPs revealed that addition of GM-CSF did not enhance immunostimulation [77]. Future avenues for the improvement of peptide vaccines include the use of modified adjuvants or long peptides, the inclusion of new antigens and combination therapy with other immunologically active agents [78]. Furthermore, optimization of the GM-CSF dose may be important for inducing immunogenicity. There is evidence that repeated low doses of GM-CSF (40–80 μg for 1–5 days) can enhance vaccine-induced immune responses, but a detrimental effect is observed at higher doses (100–500 μg for 1–5 days) [43]. This may be due to activation of anti-tumor responses at low doses, with higher doses inducing mobilization of MDSCs from the bone marrow and causing subsequent immune suppression [43]. Most studies of peptide vaccines evaluated here used a relatively high dose of GM-CSF, which may not be optimal, and they did not investigate MDSC activation. Overall, GM-CSF was well tolerated and did not have a detrimental effect on outcomes.

Generation of anti-GM-CSF antibodies could limit the activity of GM-CSF in vaccines and other treatments. Mice treated with adenoviruses and plasmids engineered to express GM-CSF produced neutralizing antibodies against GM-CSF that were associated with suppressed CD8+ T-cell responses [79]. When GM-CSF was given to patients with metastatic colorectal cancer, 95 % (19 of 20) developed anti-GM-CSF antibodies. In a subset of those patients, development of high-titer anti-GM-CSF responses was accompanied by a significant reduction in GM-CSF-induced granulocytes, although the number of granulocytes did not decrease below baseline values [80]. More recently, a patient with glioblastoma developed grade 3 toxicity following treatment with a DC vaccine in combination with GM-CSF. This sensitization was associated with the production of anti-GM-CSF antibodies and was reversed when GM-CSF treatment was stopped [81]. Although anti-GM-CSF antibody production is rarely associated with clinical symptoms, it may reduce the efficacy of GM-CSF in immunotherapy. Therefore, monitoring the development of anti-GM-CSF antibodies during clinical trials of GM-CSF should be considered. Spontaneous development of anti-GM-CSF antibodies (in the absence of exogenous GM-CSF) has been observed [82], and this should be taken into consideration when analyzing treatment-induced anti-GM-CSF antibodies.

The use of GM-CSF as an adjuvant to surgery has shown some clinical benefit, including improvements in OS [54, 55]. Although most patients in these studies were classified as having stage III–IV disease, they were considered to be disease-free at the time of study entry. Therefore, this population may be different from those who are not suitable for surgery, in that they were unlikely to have metastatic disease. Nonetheless, in patients with stage IIIC disease, MSS was significantly improved in patients who received GM-CSF following complete surgical resection compared with those who did not receive GM-CSF [47]. Since the completion of this systematic review, a double-blind randomized placebo-controlled phase 3 study has been published in which patients with stage IV or high-risk stage III disease who had undergone surgical resection did not have significantly improved survival following treatment with a peptide vaccine, with or without GM-CSF, compared with placebo [83]. However, exploratory analyses revealed a trend toward improved OS in patients with resected visceral metastases who received GM-CSF compared with those who received placebo [83]. These results suggest that GM-CSF may be effective as an adjuvant treatment to surgery in certain patients; this warrants further investigation.

Overall, GM-CSF in combination with peptide vaccines appears insufficient to induce an immune response that correlates with anti-tumor activity, whereas GM-CSF in combination with chemotherapy [56, 58, 59], autologous melanoma cell vaccine [66], ipilimumab [49] or recombinant vaccinia virus [65] has shown induction of immune responses, together with evidence of some clinical activity, suggesting that use of GM-CSF may be beneficial in combination with agents that stimulate the immune system sufficiently to attack tumor cells.

This systematic review has several limitations. Few studies made direct comparisons between GM-CSF and other treatments, and most involved the use of GM-CSF as an adjuvant to other therapies. Therefore, it was often difficult to distinguish between the effect of the treatment and the effect of GM-CSF on clinical and immunological outcomes. Wide ranges of GM-CSF doses, treatment regimens and comparators were evaluated in the included studies. Establishing the optimal dose of GM-CSF for adjuvant treatment is important to achieve the maximum response [43], and the range of dosing regimens employed by the studies reviewed here suggests that this is yet to be identified. There were also differences in the methodologies used to assess patient outcomes; hence, cross-study comparisons should be made with caution. The treatment groups were often small, making it difficult to extrapolate the results to the wider population of patients with advanced melanoma. Furthermore, GM-CSF is not approved for the treatment of patients with melanoma, so the patient populations described here are likely to be highly selected and may not be representative of those in clinical practice.

In conclusion, it is clear that GM-CSF has been used as an adjuvant in many different clinical trial settings; however, evidence for clinical efficacy is controversial. Some clinical benefit has been observed in patients who received GM-CSF in combination with surgery, chemotherapy or immunomodulatory agents. In general, however, outcomes for patients receiving peptide vaccines were not improved when GM-CSF was used as an adjuvant. GM-CSF is a promising therapeutic adjuvant, but there is a lack of controlled phase 3 trials investigating the direct effects of GM-CSF in patients with advanced melanoma. Small, single-center studies conducted mostly in academic institutions have not led to the design and implementation of well-controlled phase 3 clinical studies. The results presented here indicate that further studies are needed to identify the optimal treatment regimen and effectiveness of GM-CSF in patients with advanced melanoma.

### Electronic supplementary material

Below is the link to the electronic supplementary material.

Supplementary material 1 (PDF 31 kb)

## Abbreviations

ANC: Absolute neutrophil count
CI: Confidence interval
CR: Complete response
DTH: Delayed-type hypersensitivity
DTIC: Dacarbazine
gp100: Glycoprotein 100
MART-1: Melanoma antigen recognized by T cells 1
MFG-E8: Milk fat globule epidermal growth factor 8
MHP: Melanoma helper peptide
MP: Melanoma peptide
MSS: Melanoma-specific survival
NED: No evidence of disease
NR: Not reached
ORR: Overall response rate
PD: Progressive disease
PFS: Progression-free survival
PRISMA: Preferred reporting items for systematic reviews and meta-analyses
Q2W/Q3W/Q4W: Every 2/3/4 weeks
TRP-2: Tyrosine-related protein 2
