# Supplementary material for: Systematic review of the use of granulocyte–macrophage colony-stimulating factor in patients with advanced melanoma
Source: Cancer Immunol Immunother. 2016 Jul 2;65(9):1015–34. doi: 10.1007/s00262-016-1860-3 (PMC4995227; doi:10.1007/s00262-016-1860-3)
Supplement: Supplementary file 1 — Supplementary material 1 (PDF 31 kb) [file 262_2016_1860_MOESM1_ESM.pdf]

Search strings used to retrieve relevant studies from (a) PubMed, (b) Embase and (c) congress abstracts

a) PubMed search strategy

("granulocyte-macrophage colony-stimulating factor"[MeSH Terms] OR ("granulocyte-macrophage"[All Fields] AND "colony-stimulating"[All Fields] AND "factor"[All Fields]) OR ("granulocyte-macrophage colony-stimulating factor"[All Fields]) OR ("gm"[All Fields] AND "csf"[All Fields]) OR "gm csf"[All Fields] OR “GM-CSF”[All Fields] OR “GMCSF”[All Fields]) AND ("melanoma"[MeSH Terms] OR "melanoma"[All Fields])

Limits: Human; English language; publication date 01/01/2000–01/05/2015

b) Embase search strategy

#	Search
1	granulocyte macrophage colony stimulating factor/
2	GM-CSF.mp.
3	granulocyte-macrophage.mp.
4	colony-stimulating.mp.
5	factor.mp.
6	3 and 4 and 5
7	gm.mp.
8	csf.mp.
9	7 and 8
10	gm csf.mp.
11	gmcsf.mp.
12	melanoma/or metastatic melanoma/ or melanoma skin cancer/or cutaneous melanoma/or melanoma.mp. or melanoma vaccine/or melanoma metastasis
13	1 or 6
14	9 or 13
15	10 or 11 or 14
16	2 or 15
17	12 and 16
18	limit 17 to (human and English language and yr="2000 –May 2015" and (adult <18 to 64 years> or aged <65+ years>))

c) Search terms used to interrogate congress proceedings

Granulocyte-macrophage colony-stimulating factor, GM-CSF, melanoma
